# Incidence of multimorbidity and associated factors during the COVID-19 pandemic in Brazil: a cohort study

**DOI:** 10.1590/1516-3180.2021.0518.R1.15092021

**Published:** 2022-04-02

**Authors:** Felipe Mendes Delpino, Eduardo Lucia Caputo, Marcelo Cozzensa da Silva, Felipe Fossati Reichert, Bruno Pereira Nunes, Natan Feter, Jayne Santos Leite, Júlia Cassuriaga, Caroline Malue Huckembeck, Ricardo Alt, Airton José Rombaldi

**Affiliations:** I MSc. Nutritionist and Doctoral Student, Postgraduate Nursing Program, Universidade Federal de Pelotas (UFPel), Pelotas (RS), Brazil.; II MSc, PhD. Postdoctoral Research Fellow, Postgraduate Physical Education Program, Universidade Federal de Pelotas (UFPel), Pelotas (RS), Brazil.; III MSc, PhD. Associate Professor, Postgraduate Physical Education Program, Universidade Federal de Pelotas (UFPel), Pelotas (RS), Brazil.; IV MSc, PhD. Associate Professor, Postgraduate Physical Education Program, Universidade Federal de Pelotas (UFPel), Pelotas (RS), Brazil.; V MSc, PhD. Adjunct Professor, Postgraduate Nursing Program, School of Nursing, Universidade Federal de Pelotas (UFPel), Pelotas (RS), Brazil.; VI PhD. Research Collaborator, Postgraduate Physical Education Program, Universidade Federal de Pelotas (UFPel), Pelotas (RS), Brazil.; VII MSc. Doctoral Student, Postgraduate Health Sciences Program, Universidade Federal do Rio Grande do Sul (UFRGS), Porto Alegre (RS), Brazil.; VIII Master’s Student, Postgraduate Physical Education Program, Universidade Federal de Pelotas (UFPel), Pelotas (RS), Brazil.; IX Master’s Student, Postgraduate Physical Education Program, Universidade Federal de Pelotas (UFPel), Pelotas (RS), Brazil.; X Master’s Student, Postgraduate Epidemiology Program, Universidade Federal de Pelotas (UFPel), Pelotas (RS), Brazil.; XI MSc, PhD. Full Professor, Postgraduate Physical Education Program, Universidade Federal de Pelotas, (UFPel), Pelotas (RS), Brazil.

**Keywords:** Multimorbidity, Chronic disease, Risk factors, Pandemics, COVID-19, Incidence of multimorbidity, Chronic illness, Coronavirus disease 2019

## Abstract

**BACKGROUND:**

Due to the coronavirus disease 2019 (COVID-19) pandemic, access to healthcare services may have become difficult, which may have led to an increase in chronic diseases and multimorbidity.

**OBJECTIVES:**

To assess the incidence of multimorbidity and its associated factors among adults living in the state of Rio Grande do Sul, Brazil.

**DESIGN AND SETTING:**

Cohort study conducted in Rio Grande do Sul, Brazil.

**METHODS:**

We included data from the two waves of the Prospective Study About Mental and Physical Health (PAMPA). Data were collected via online questionnaires between June and July 2020 (wave 1) and between December 2020 and January 2021 (wave 2). Multimorbidity was defined as the presence of two or more diagnosed medical conditions.

**RESULTS:**

In total, 516 individuals were included, among whom 27.1% (95% confidence interval, CI: 23.5-31.1) developed multimorbidity from wave 1 to 2. In adjusted regression models, female sex (hazard ratio, HR: 1.97; 95% CI: 1.19-3.24), middle-aged adults (31-59 years) (HR: 1.78; 95% CI: 1.18-2.70) and older adults (60 or over) (HR: 2.41; 95% CI: 1.25-4.61) showed higher risk of multimorbidity. Back pain (19.4%), high cholesterol (13.3%) and depression (12.2%) were the medical conditions with the highest proportions reported by the participants during wave 2.

**CONCLUSION:**

The incidence of multimorbidity during a six-month period during the COVID-19 pandemic was 27.1% in the state of Rio Grande do Sul, Brazil.

## INTRODUCTION

Multimorbidity, defined as the presence of two or more chronic diseases, is associated with reduced quality of life.^1^ Globally, multimorbidity affects one in three people, although this prevalence might be higher among women and older adults.^2^ In Brazil, the prevalence of multimorbidity may reach up to 24% among adults.^3,4^ However, the prevalence is even higher among older adults in Brazil, such that in 2015 it was found to be affecting at least half of this population.^5,6^

The elevated costs attributable to multimorbidity treatment are alarming. For example, the average medical economic cost of multimorbidity is 5.5 times greater than the treatment of only one chronic condition in Switzerland.^7^ Each additional disease represents a 3.2-fold increase in requirement of healthcare services and roughly 33% greater treatment costs.^7^ Multimorbidity is also associated with increased use of healthcare resources including medications, primary care visits, hospitalizations, elective procedures and emergency services.^8^ In Brazil, a study carried out using data from the Brazilian National Health Survey (Pesquisa Nacional de Saúde, PNS) showed that patients with multimorbidity were more likely to use healthcare services.^9^

Nevertheless, treatments or diagnoses for multimorbidity may have become impaired due to the limitations on access to the healthcare system that occurred as an indirect consequence of the coronavirus disease 2019 (COVID-19) pandemic.^10^ In December 2020, Brazil had the second-highest number of registered cases and deaths due to COVID-19.^11^ This chaotic scenario limited the management of preexisting chronic disease, especially because of fear of viral contagion during medical appointments.^10^ Furthermore, diagnosing of other morbidities may have been equally impaired during social distancing. Besides the higher risk of severe COVID-19 associated with the presence of chronic disease, co-occurrence of different morbidities might increase the risk of COVID-19 complications, including hospitalization. However, to the best of our knowledge, longitudinal studies that examine the incidence of multimorbidity during the COVID-19 pandemic remain warranted.

## OBJECTIVE

The aim of this study was to evaluate the incidence of multimorbidity and its associated factors during the COVID-19 pandemic, among adults living in the state of Rio Grande do Sul, Brazil.

## METHODS

We analyzed data from waves 1 and 2 of the Prospective Study about Mental and Physical Health (PAMPA) cohort, which was an ambispective study carried out in the state of Rio Grande do Sul, Brazil. In wave 1, the recruitment phase took place between June 22 and July 23, 2020, while wave 2 was carried out between December 1, 2020, and January 15, 2021. Wave 2 lasted longer (seven weeks) due to the holiday period. Full details of the study methodology can be found elsewhere.^12^ The study protocol was approved (number: 4.093.170; date: August 27, 2019) by the institutional research ethics board of the Superior School of Physical Education of the Universidade Federal de Pelotas (UFPel), Brazil. All participants gave informed consent before answering any question from the questionnaire.

### Recruitment phase

During the recruitment phase, we contacted participants through university professors, social media, local media and personal contacts in all macroregions in the state. Only adults aged 18 or older and living in Rio Grande do Sul were included in wave 1. We also contacted the local media in all macroregions to inform the local population about the present study. Moreover, all researchers involved shared the link to the study announcement with their personal contacts across the state. For wave 2, we contacted the previous participants who were still living in the state and had provided any contact information at the time of wave 1 (e.g. phone number or social media nickname). The questionnaires were constructed using the Google Forms application in wave 1 (Google, Mountain View, California, United States) and using the Redcap web application, version 9.0.3, in wave 2 (Vanderbilt University, Nashville, Tennessee, United States).

### Sample size

We calculated the sample size based on three primary outcomes: low back pain; depressive and anxiety symptoms; and access to the healthcare system.^12^ The total population of the state of Rio Grande do Sul was 10,693,929 in 2010, according to the 2010 Brazilian national census. We defined that a sample of 1,767 participants was required, under the assumptions of a 95% confidence interval, a margin of error of 1.8, and a possible loss-to-follow-up of 30%. Rio Grande do Sul is divided into seven macroregions named (in Portuguese): Serra, Norte, Nordeste, Centro-Oeste, Vales, Metropolitana and Sul. Based on the latest national census, the required sample size was divided proportionally to the number of people living in each region.

### Outcome

In this study, we excluded participants who presented multimorbidity in wave 1. Multimorbidity was assessed through the same question previously used in the Brazilian Telephone-based Surveillance System for Noncommunicable Diseases:^13^ “*Has any doctor ever told you that you have the following disease?*”. A 12-item list was used, which included the following diseases: hypertension or high blood pressure, diabetes, high cholesterol, cancer, arthritis/arthrosis/fibromyalgia, asthma/bronchitis, back problem, heart disease, depression, memory problem, human immunodeficiency virus (HIV)/acquired immunodeficiency syndrome (AIDS) and other chronic diseases. Multimorbidity was considered to be present when a participant reported having two or more health issues.^14^

### Exposures

Sociodemographic factors (age, sex, ethnicity, conjugal situation and educational level), behavioral information (physical activity) and nutritional data (body mass index, BMI) were used as possible confounders and as associated factors.

Weight and height were self-reported and were used to calculate BMI (kg/m^2^). We considered subjects to be overweight if they had BMI > 25 kg/m^2^ and obese if they had BMI ≥ 30 kg/m^2^. To estimate what the subjects’ physical activity level had been before social distancing, we used the following question: “*Before social distancing, were you engaged in physical activity regularly?*”. If the participants answered “Yes”, they were asked to declare the number of days with physical activity and its duration in minutes, in a regular pre-COVID-19 week. Participants who practiced for 150 minutes or more were classified as physically active, and those who practiced for less than 150 minutes weekly, as physically inactive, in accordance with the guidelines for physical activity from the World Health Organization.^15^

Decreased monthly income was assessed by the following question: “*Over the last six months, have your monthly earnings been affected by social distancing measures?*”. There were three response options: “decreased”, “unchanged” “increased”. We classified this into a dichotomous variable for the analyses, with the option “yes” for decreased monthly income and “no” for unaffected or increased income.

### Data analyses

**Figure 1.**
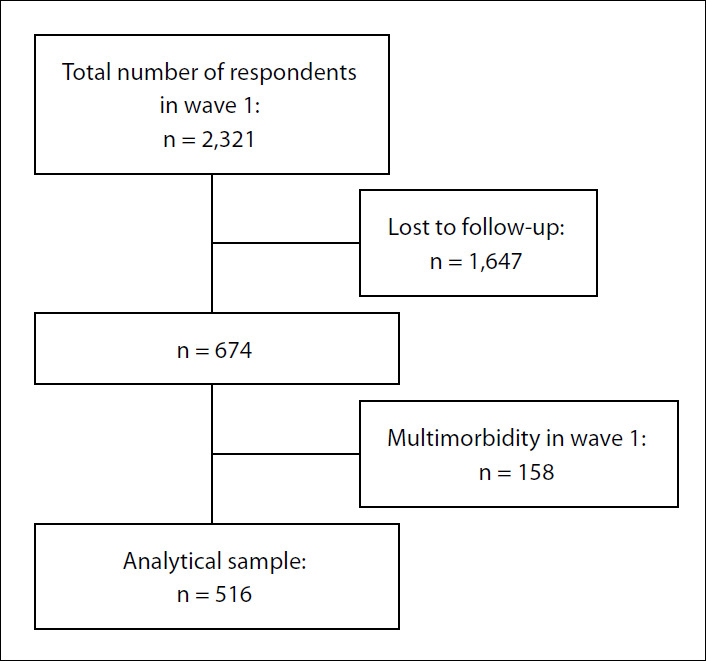
Flow chart describing sampling process.

All analyses were weighted according to the proportion of the participants in each macroregion, because of overrepresentation of respondents living in the Sul region (n = 436; 64.6%). The data were reported as the mean with 95% confidence interval (CI) or as proportions with 95% CI, as appropriate. Participants who provided some personal contact in wave 1 but did not respond to wave 2 were excluded (Figure 1). We used proportional-hazards global tests and visual examination of Schoenfeld residuals against time to assess proportional-hazards assumptions (data not shown). Crude and adjusted Cox regression models were used to estimate the hazard ratio (HR) with a 95% CI.^16^ The adjusted model (aHR) included sex, age, ethnicity, conjugal situation, education level, decreased monthly income, BMI and physical activity before the pandemic. All covariates were assessed at wave 1. Pearson’s chi-square test was used to test the differences between included and excluded samples (Table 1). Analyses were performed using the Stata 15.1 software (Stata Corp, College Station, Texas, United States).

**Table 1.** Sociodemographic and behavioral characteristics of the included and lost-to-follow-up participants. Rio Grande do Sul, Brazil (n = 516)

	Included sample %	Lost to follow-up %	P-value
**Sex**			0.149
Male	21.5	22.7	
Female	78.5	77.3	
**Age (years)**			0.370
18-30	42.7	28.0	
31-59	49.9	60.0	
60+	7.4	12.0	
**Skin color**			0.409
White	90.3	87.8	
Black	5.5	7.0	
Mixed	3.7	4.8	
Other	0.04	0.4	
**Marital status**			0.912
Living with a partner	57.2	64.3	
Living alone	42.8	35.7	
**Education level**			0.306
High school or lower	33.8	37.4	
University degree	23.5	25.1	
Specialization, Master’s, PhD	42.7	37.5	
**Decreased monthly income**			0.475
No	66.0	53.7	
Yes	34.0	46.3	
**Nutritional status**			0.820
Normal	51.7	41.2	
Overweight	32.3	35.0	
Obese	16.0	23.7	
**Physical activity before COVID-19 pandemic**			0.262
Inactive	57.0	58.6	
Active	43.0	41.4	

Based on body mass index, calculated from self-reported height and body weight. COVID-19 = coronavirus disease 2019.

## RESULTS

A flowchart describing the sampling process is presented in Figure 1. Out of 2,321 participants with valid responses in wave 1, 1,647 were lost during follow-up. Thus, a final sample of 674 participants was included in wave 2. However, 23% (95% CI: 19.9-28.3) were excluded from the present analysis because they reported having multimorbidity in wave 1. Consequently, 516 participants were eligible for this study.

The main characteristics of the sample are described in Table 1. Most of the participants were women (78.5%; 95% CI: 73.3-80.83), white (90.3%; 95% CI: 86.5-93.2) and lived with a partner (57.2%; 95% CI: 51.5-62.8). Almost half of the participants were aged between 31 and 59 years (49.9%; 95% CI: 44.2-55.6) and 23.5% (95% CI: 19.1-28.6) had at least one academic degree. Comparison of the excluded and lost-to-follow-up sample with the sample included from the second wave showed that none of the variables differed according to the chi-square test.

Overall, 27% (95% CI: 23.5-31.1) of the participants presented with new cases of multimorbidity. The risk of incident multimorbidity according to sociodemographic, behavioral and nutritional characteristics is presented in Table 2. Women (aHR: 1.97; 95% CI: 1.19-3.24) and subjects aged 31-59 years (aHR: 1.78; 95% CI: 1.18-2.70) and aged 60 years or over (aHR: 2.41; 95% CI: 1.25-4.61) were more likely to have incident multimorbidity. Even though BMI (obesity) and physical activity showed significant associations with multimorbidity in the crude analysis, no significant results were observed in the adjusted analyses (aHR: 1.54; 95% CI: 0.97-2.44; aHR: 0.68; 95% CI: 0.46-1.01, respectively).

**Table 2.** Hazard ratios for multimorbidity according to single lifestyle factors among participants in the Prospective Study About Mental and Physical Health (PAMPA) Cohort, Rio Grande do Sul, Brazil (n = 516)

	Crude hazard ratio (95% CI)	P-value	Adjusted hazard ratio^1^ (95% CI)	P-value
**Sex**		< 0.001*		0.008*
Male	1.00		1.00	
Female	1.85 (1.14; 3.0)		1.97 (1.19; 3.24)	
**Age (years)**		< 0.001*		< 0.001*
18-30	1.00		1.00	
31-59	1.85 (1.27; 2.68)		1.78 (1.18; 2.70)	
60+	2.42 (1.32; 4.44)		2.41 (1.25; 4.61)	
**Skin color**		0.907		0.633
White	1.00		1.00	
Mixed	1.5 (0.83; 2.71)		1.82 (0.98; 3.37)	
Black	0.53 (0.17; 1.68)		0.53 (0.17; 1.70)	
Other	3.74 (0.52; 26.8)		4.11 (0.55; 30.7)	
**Marital status**		0.848		0.208
Living with a partner	1.00		1.00	
Living alone	1.03 (0.74; 1.45)		1.31 (0.92; 1.88)	
**Education level**		0.161		0.618
High school or lower	1.00		1.00	
University degree	1.11 (0.70; 1.76)		0.99 (0.62; 1.61)	
Specialization, Master’s, PhD	1.31 (0.89; 1.95)		1.05 (0.68; 1.64)	
**Decreased monthly income**		0.736		0.489
No	1.00		1.00	
Yes	0.75 (0.53; 1.06)		0.83 (0.58; 1.19)	
**Nutritional status**		0.031*		0.081
Normal	1.00		1.00	
Overweight	1.37 (0.94; 1.98)		1.33 (0.90; 1.96)	
Obese	1.57 (1.01; 2.45)		1.54 (0.97; 2.44)	
**Physical activity before COVID-19 pandemic**		0.020*		0.066
Inactive	1.00		1.00	
Active	0.65 (0.45; 0.95)		0.68 (0.46; 1.01)	

*Statistically significant P-values; ^1^Adjusted for sex, age, skin color, conjugal situation, education level, decreased monthly income, body mass index (BMI) and physical activity before coronavirus disease 2019 (COVID-19) pandemic, at baseline. CI = confidence interval.

**Figure 2.**
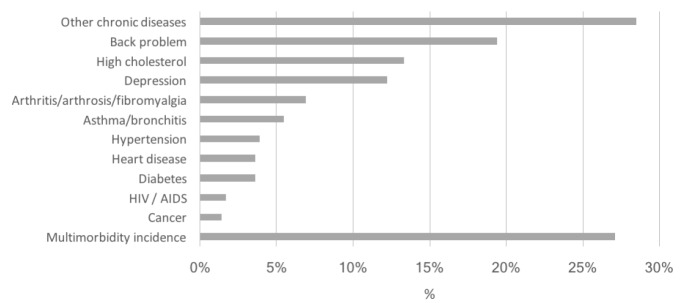
Multimorbidity incidence and list of the new diseases reported between the first and second waves.

The list of diseases and their frequencies are presented in Figure 2. The incidence of multimorbidity was 27.1% (95% CI: 23.5-31.1). The most common new diseases reported between waves 1 and 2 were among those in our initial 12-item list (28.5%). Back pain represented 19.4% of the new cases, followed by high cholesterol (13.3%) and depression (12.2%). There were no new occurrences of memory problems during the pandemic.

There were no new occurrences of memory problems. HIV = human immunodeficiency virus; AIDS = acquired immunodeficiency syndrome.

## DISCUSSION

About one in four participants developed multimorbidity between waves 1 and 2 (six months). Women and adults aged 31 years or over showed higher risk of incident multimorbidity. We observed a dose-response relationship between age and the risk of multimorbidity, in which the risk increased with increasing age.

Female sex was associated with higher risk of incident multimorbidity during the period of COVID-19 social distancing, compared with males. Previously, other studies had shown that women were twice as likely to report multimorbidity as were men, both in Brazil and in other countries.^2,4,17,18^ Greater frequency of use of healthcare systems through medical appointments among women might explain this relationship. On the other hand, men might be more likely to be underdiagnosed with regard to chronic conditions and multimorbidity.^19^ Furthermore, women experience more stressful events throughout their lives^20^ and therefore are at higher risk of chronic diseases. Unfortunately, impairment of mental health during social distancing seems to be higher among women than among men.^21^ Furthermore, we previously showed that access to the healthcare system during social distancing was more impaired among women.^10^ Therefore, strategies to improve healthcare system access are required, in order to monitor the prevalence and incidence of chronic diseases, especially among women.

In Brazil, previous studies also showed that older adults, especially those over 60 years of age, had higher prevalence of multimorbidity than younger adults.^4,18^ A systematic review from 2019 showed that individuals aged 65 or more were the most affected by multimorbidity, worldwide.^2^ Although population aging might be associated with this increased prevalence, previous studies have suggested other factors, including impaired healthcare access and low social development.^22^ For example, among older adults, multimorbidity is influenced by socioeconomic, demographic, lifestyle and family factors.^23^ Also, older people became more isolated during the pandemic,^21^ and the high number of new cases of depression may have been related to this.

The lack of statistically significant relationships with multimorbidity shown by BMI and physical activity in the adjusted analyses may have been due to the relatively short time between waves 1 and 2 (six months). For physical activity, a previous longitudinal study did not show any association with the development of multimorbidity in short-term follow-ups (two years).^24^ However, for longer follow-ups (11 years), physical activity was shown to be protective against development of multimorbidity.^25^ A previous study in Finland showed that physical inactivity and obesity were risk factors for incident multimorbidity. However, that study included only five chronic conditions in the multimorbidity list.^26^ According to our results, being physically active before the pandemic did not protect against developing multimorbidity during the pandemic. Despite this, physical activity is important and needs to be encouraged. For instance, physical activity in the early ages of childhood plays a protective role against multimorbidity at older ages.^27^

The COVID-19 pandemic affected access to healthcare services, especially for chronic diseases like diabetes, chronic obstructive pulmonary disease and hypertension.^28^ Especially in low and middle-income countries, COVID-19 affected healthcare services with consequences ranging from postponement of elective surgery and other medical procedures to delayed treatment of chronic diseases that might progress to severe conditions.^29^ Our results reflected those findings, and we demonstrated that the less-reported problems before social distancing, such as back pain and depression, were the ones with higher frequency during wave 2. Moreover, a study conducted in India showed that individuals with multimorbidity experienced several care challenges, including interruption of treatment and routine check-ups during the pandemic.^30^

The increase in new cases of depression observed in our data was in agreement with the recent literature.^31^ A sharp increase in the levels of psychological distress was evident during the pandemic, especially among women and adults aged younger than 60.^31^ Moreover, a meta-analysis on community-based studies conducted during the COVID-19 pandemic showed that the prevalence of depression was seven times higher than the estimated prevalence in 2017.^32^ Thus, strategies to attenuate the detrimental indirect effects of this pandemic on mental health, especially in higher-risk groups, are warranted.

Furthermore, spending more time at home with sedentary activities and less time exercising might explain the high number of spine problems. A large number of cases of back pain occurrences may have resulted from COVID-19, specifically related to the social distancing measures that made many people start working from home without adequate equipment. Previous studies also reported similar findings, thus showing that the social distancing caused by COVID-19 resulted in more occurrences of low back pain and more associated risk factors.^33^

Our study had some methodological limitations. First, we did not include an open question for “other diseases”. Thus, it was not possible to identify which other diseases were reported by the participants. Second, our retention rate was lower than expected for face-to-face cohort studies.^12^ However, our results are similar to those of other cohort studies that were conducted online.^34^ Third, we assessed the list of diseases through self-reports, which is less accurate than use of medical records because it depends on the memory of each participant. However, most other studies also used self-reported data to assess multimorbidity, according to a systematic review conducted in 2019.^2^ Fourth, our respondents included a high proportion of individuals with academic degrees. Given that data collection was online, and university professors shared the questionnaire through their academic contacts, a high number of participants with academic degrees was expected. Also, people with less education have less access to the internet. Furthermore, according to previous findings in the literature, from samples with less schooling, the incidence of multimorbidity could have been even higher.^35^

However, we can highlight the longitudinal design of our study evaluating multimorbidity incidence during the pandemic. This may have generated important information about the development of chronic diseases in this context. Moreover, at the time when we conducted this study, we did not find any other cohort study assessing the incidence of multimorbidity during the COVID-19 pandemic. Our results may provide relevant information about the consequences of the COVID-19 pandemic on the health of the population of southern Brazil. Furthermore, our report provides important information about a new profile of people who will seek healthcare services during and after the pandemic.

## CONCLUSION

The incidence of multimorbidity over a six-month period during the COVID-19 pandemic was 27.1% in the state of Rio Grande do Sul, in Brazil. Female sex and increasing age were risk factors for incident multimorbidity. Special attention to the risk that women and older people may develop multimorbidity is needed within public health policies, along with attention to the high numbers of new cases of depression and back pain during the COVID-19 pandemic.

## References

[1] Makovski TT, Schmitz S, Zeegers MP, Stranges S, van den Akker M (2019). Multimorbidity and quality of life: Systematic literature review and meta-analysis. Ageing Res Rev..

[2] Nguyen H, Manolova G, Daskalopoulou C (2019). Prevalence of multimorbidity in community settings: A systematic review and meta-analysis of observational studies. J Comorb..

[3] Costa ÂK, Bertoldi AD, Fontanella AT (2020). Does socioeconomic inequality occur in the multimorbidity among Brazilian adults?. Rev Saude Publica..

[4] Carvalho JN, Roncalli ÂG, Cancela MC, De Souza DL (2017). Prevalence of multimorbidity in the Brazilian adult population according to socioeconomic and demographic characteristics. PLoS One..

[5] Melo LA, Lima KC (2020). Prevalence and factors associated with multimorbidities in Brazilian older adults. Cienc e Saude Coletiva..

[6] Nunes BP, Batista SRR, Andrade FB (2018). Multimorbidity: The Brazilian Longitudinal Study of Aging (ELSI-Brazil). Rev Saude Publica..

[7] Bähler C, Huber CA, Brüngger B, Reich O (2015). Multimorbidity, health care utilization and costs in an elderly community-dwelling population: A claims data based observational study. BMC Health Serv Res..

[8] McPhail SM (2016). Multimorbidity in chronic disease: Impact on health care resources and costs. Risk Manag Healthc Policy..

[9] de Souza ASS, Braga JU (2020). Trends in the use of health services and their relationship with multimorbidity in Brazil, 1998-2013. BMC Health Serv Res..

[10] Leite JS, Feter N, Caputo EL (2021). Managing noncommunicable diseases during the covid-19 pandemic in Brazil: Findings from the pampa cohort. Cienc e Saude Coletiva..

[11] World Health Organization (2020). Coronavirus Disease..

[12] Feter N, Caputo EL, Doring IR (2020). Longitudinal study about low back pain, mental health, and access to healthcare system during COVID-19 pandemic: Protocol of an ambispective cohort. medRxiv.

[13] Enes CC, Nucci LB (2019). A Telephone Surveillance System for Noncommunicable Diseases in Brazil. Public Health Rep..

[14] Valderas JM, Starfield B, Sibbald B, Salisbury C, Roland M (2009). Defining comorbidity: Implications for understanding health and health services. Ann Fam Med..

[15] Bull FC, Al-Ansari SS, Biddle S (2020). World Health Organization 2020 guidelines on physical activity and sedentary behaviour. Br J Sports Med..

[16] Barros AJ, Hirakata VN (2003). Alternatives for logistic regression in cross-sectional studies: An empirical comparison of models that directly estimate the prevalence ratio. BMC Med Res Methodol..

[17] Alimohammadian M, Majidi A, Yaseri M (2017). Multimorbidity as an important issue among women: Results of a gender difference investigation in a large population-based cross-sectional study in West Asia. BMJ Open..

[18] Nunes BP, Camargo-Figuera FA, Guttier M (2016). Multimorbidity in adults from a southern Brazilian city: occurrence and patterns. Int J Public Health..

[19] Bertakis KD, Azari R, Helms LJ, Callahan EJ, Robbins JA (2000). Gender Differences in the Utilization of Health Care Services. J Fam Pract..

[20] Mauvais-Jarvis F, Bairey Merz N, Barnes PJ (2020). Sex and gender: modifiers of health, disease, and medicine. The Lancet..

[21] Batista SR, Souza ASS, Nogueira J (2020). Protective behaviors for COVID-19 among Brazilian adults and elderly living with multimorbidity: The ELSI-COVID-19 initiative. Cad Saude Publica..

[22] van Oostrom SH, Gijsen R, Stirbu I (2016). Time trends in prevalence of chronic diseases and multimorbidity not only due to aging: Data from general practices and health surveys. PLoS One..

[23] Melo LA, Braga LC, Leite FPP (2019). Factors associated with multimorbidity in the elderly: an integrative literature review. Rev Bras Geriatr e Gerontol..

[24] Ryan A, Murphy C, Boland F, Galvin R, Smith SM (2018). What is the impact of physical activity and physical function on the development of multimorbidity in older adults over time? A population-based cohort study. J Gerontol A Biol Sci Med Sci..

[25] Dhalwani NN, O’Donovan G, Zaccardi F (2016). Long terms trends of multimorbidity and association with physical activity in older English population. Int J Behav Nutr Phys Act..

[26] Wikström K, Lindström J, Harald K, Peltonen M, Laatikainen T (2015). Clinical and lifestyle-related risk factors for incident multimorbidity: 10-year follow-up of Finnish population-based cohorts 1982-2012. Eur J Intern Med..

[27] Feter N, Leite JS, Umpierre D, Caputo EL, Rombaldi AJ (2021). Multimorbidity and leisure-time physical activity over the life course: a population-based birth cohort study. BMC Public Health..

[28] Chudasama YV, Gillies CL, Zaccardi F (2020). Impact of COVID-19 on routine care for chronic diseases: A global survey of views from healthcare professionals. Diabetes Metab Syndr..

[29] Okereke M, Ukor NA, Adebisi YA (2021). Impact of COVID-19 on access to healthcare in low- and middle-income countries: Current evidence and future recommendations. Int J Health Plann Manage..

[30] Pati S, Mahapatra P, Kanungo S, Uddin A, Sahoo KC (2021). Managing Multimorbidity (Multiple Chronic Diseases) Amid COVID-19 Pandemic: A Community Based Study From Odisha, India. Front Public Health..

[31] Xiong J, Lipsitz O, Nasri F, Lui LMW, Gill H, Phan L (2020). Impact of COVID-19 pandemic on mental health in the general population: A systematic review. J Affect Disord..

[32] Bueno-Notivol J, Gracia-García P, Olaya B (2021). Prevalence of depression during the COVID-19 outbreak: A meta-analysis of community-based studies. Int J Clin Health Psychol..

[33] Šagát P, Bartík P, González PP, Tohănean DI, Knjaz D (2020). Impact of COVID-19 quarantine on low back pain intensity, prevalence, and associated risk factors among adult citizens residing in Riyadh (Saudi Arabia): A cross-sectional study. Int J Environ Res Public Health..

[34] Brown M, Goodman A, Peters A, Ploubidis GB (2021). COVID-19 Survey in Five National Longitudinal Studies: Wave 1, 2 and 3 User Guide (Version 3)..

[35] Costa CDS, Flores TR, Wendt A (2018). Inequalities in multimorbidity among elderly: A population-based study in a city in Southern Brazil. Cad Saude Publica..

